# Real-time non-invasive hemoglobin prediction using deep learning-enabled smartphone imaging

**DOI:** 10.1186/s12911-024-02585-1

**Published:** 2024-07-01

**Authors:** Yuwen Chen, Xiaoyan Hu, Yiziting Zhu, Xiang Liu, Bin Yi

**Affiliations:** 1 Chongqing Institute of Green and Intelligent Technology, Chinese Academy of Sciences, Chongqing, 400714 China; 2 Department of Anaesthesiology, Southwest Hospital, Third Military Medical University, Chongqing, 400038 China

**Keywords:** Non-invasive prediction, Smartphone, Hemoglobin, Deep learning, Automatic

## Abstract

**Background:**

Accurate measurement of hemoglobin concentration is essential for various medical scenarios, including preoperative evaluations and determining blood loss. Traditional invasive methods are inconvenient and not suitable for rapid, point-of-care testing. Moreover, current models, due to their complex parameters, are not well-suited for mobile medical settings, which limits the ability to conduct frequent and rapid testing. This study aims to introduce a novel, compact, and efficient system that leverages deep learning and smartphone technology to accurately estimate hemoglobin levels, thereby facilitating rapid and accessible medical assessments.

**Methods:**

The study employed a smartphone application to capture images of the eye, which were subsequently analyzed by a deep neural network trained on data from invasive blood test data. Specifically, the EGE-Unet model was utilized for eyelid segmentation, while the DHA(C3AE) model was employed for hemoglobin level prediction. The performance of the EGE-Unet was evaluated using statistical metrics including mean intersection over union (MIOU), F1 Score, accuracy, specificity, and sensitivity. The DHA(C3AE) model’s performance was assessed using mean absolute error (MAE), mean-square error (MSE), root mean square error (RMSE), and R^2.

**Results:**

The EGE-Unet model demonstrated robust performance in eyelid segmentation, achieving an MIOU of 0.78, an F1 Score of 0.87, an accuracy of 0.97, a specificity of 0.98, and a sensitivity of 0.86. The DHA(C3AE) model for hemoglobin level prediction yielded promising outcomes with an MAE of 1.34, an MSE of 2.85, an RMSE of 1.69, and an R^2 of 0.34. The overall size of the model is modest at 1.08 M, with a computational complexity of 0.12 FLOPs (G).

**Conclusions:**

This system presents a groundbreaking approach that eliminates the need for supplementary devices, providing a cost-effective, swift, and accurate method for healthcare professionals to enhance treatment planning and improve patient care in perioperative environments. The proposed system has the potential to enable frequent and rapid testing of hemoglobin levels, which can be particularly beneficial in mobile medical settings.

**Trial Registration:**

The clinical trial was registered on the Chinese Clinical Trial Registry (No. ChiCTR2100044138) on 20/02/2021.

## Introduction

Hemoglobin, a critical oxygen-carrying protein in the blood, is a central biomarker for diagnosing and managing an array of medical conditions. The monitoring of hemoglobin levels is of utmost importance in clinical practice, playing a critical role in several key areas. Firstly, it is essential in diagnosing and treating anemia, as hemoglobin levels directly reflect the presence and severity of red blood cell deficiencies. Secondly, hemoglobin measurement aids in assessing the risk of cardiovascular diseases, given its association with blood viscosity and oxygen delivery to vital organs. Furthermore, preoperative evaluations rely heavily on hemoglobin levels to ensure patients’ hemodynamic stability during surgical procedures. Finally, accurate quantification of blood loss is crucial in trauma and surgical settings, and hemoglobin monitoring provides a timely indicator of such losses. The clinical significance of hemoglobin measurement underscores the need for accurate, reliable, and accessible testing methods. Accurate hemoglobin measurement is not only vital for diagnosing and managing medical conditions but also for guiding critical clinical decisions, such as determining transfusion requirements, thereby optimizing patient care and outcomes. Traditional methods of measuring hemoglobin, predominantly based on invasive blood tests, often result in patient discomfort and are time-consuming. This poses challenges in scenarios where rapid and non-invasive assessment is desirable. This not only poses challenges in emergency or mobile medical settings where rapid assessment is crucial but also limits the frequency of testing due to the discomfort and risk of infection associated with invasive procedures. The quest for non-invasive alternatives has led to the development of non-invasive techniques to gauge hemoglobin levels [1, 2], including pulse oximetry and spectrophotometry. These methods, which measure oxygen saturation and the absorption of light by hemoglobin respectively, however, come with their limitations. Factors such as skin color, temperature, and motion artifacts can influence their accuracy.

The emergence of smartphone technology has paved the way for a novel approach to estimating hemoglobin levels. Leveraging deep learning algorithms, smartphone-based systems are now capable of predicting hemoglobin concentrations [3, 4]. These systems harness the power of smartphones’ built-in sensors, such as cameras and flashlights, to capture skin images for analysis via deep learning algorithms. Artificial intelligence (AI) has the potential to significantly enhance the precision and reliability of these sensors while simultaneously reducing detection costs and time. Given their convenience, accessibility, and cost-effectiveness, smartphone-based diagnostic methods are revolutionizing medical detection, offering clinicians improved diagnostic tools.

This paper introduces a pioneering system that utilizes smartphones, coupled with deep learning algorithms, to predict hemoglobin concentration accurately. By integrating clinical data from patients’ eyelids and employing a streamlined network architecture, this system can ascertain hemoglobin levels without external equipment. Moreover, it achieves higher accuracy in real-time detection than manual assessments conducted by medical professionals. This advancement represents a significant stride toward more accessible and efficient healthcare diagnostics, promising enhanced patient care and outcomes.

Our main contributions in this work are as follows: 1) The development of a non-invasive hemoglobin measurement system using smartphone imaging, addressing the need for a convenient and rapid testing method; 2) The implementation and evaluation of the EGE-Unet model for eyelid segmentation, showcasing its superior performance over existing models in terms of accuracy and efficiency; 3) The application of the DHA(C3AE) model for hemoglobin level prediction, demonstrating its effectiveness in providing accurate and reliable results; 4) The innovation of a streamlined deep learning framework suitable for mobile deployment, with a focus on computational efficiency and practicality.

The remainder of this paper is organized as follows: The next section provides a comprehensive review of the literature related to non-invasive hemoglobin measurement and the application of deep learning in smartphone-based health diagnostics. This is followed by the Methods section, where we detail our experimental setup, participant recruitment, data acquisition, and the deep learning model architecture. The Results section presents the performance of our system, including accuracy and reliability assessments. We then discuss the implications of our findings in the Discussion section, comparing our results with existing literature and exploring potential clinical applications. Finally, the paper concludes with a summary of our contributions and an outlook on future research directions.

### Related work

Several approaches have been explored to determine hemoglobin (Hb) concentrations from blood specimens [5–9]. Traditional machine learning models, utilizing invasive methods, have been frequently employed for Hb concentration prediction [7–9]. While these models require blood samples obtained through venipuncture and rely on costly, specialized optical measurement equipment, they offer high precision in their results. In the field of medical image processing, researchers have utilized various techniques to address diagnoses and predictions of eye-related diseases, including deep learning and hybrid algorithm for machine learning [10–15]. Similarly, recent advancements have introduced non-invasive techniques for Hb prediction, leveraging image processing and machine learning [16–18]. Notably, deep learning methodologies utilizing images of the fingertip and eye have been applied to categorize Hb levels [19, 20]. One particular study [21] achieved a rank order correlation of 0.93 between its predictions and actual Hb levels using fingertip videos processed by an Artificial Neural Network. Another research [22] effort utilized a Convolutional Neural Network (CNN) analyzing eye images for anemia classification, achieving an impressive accuracy rate of 94%. Additional techniques [23] for Hb level estimation have employed facial feature extraction alongside Inception V3 for classification, as well as the analysis of the near-infrared spectrum of spent dialysis fluid to predict anemia [24].

Despite these innovations, non-invasive quantification of Hb levels remains an area with limited exploration. A noteworthy [20] attempt involved quantifying Hb concentration through non-invasive means using fundus images for training, although this method presented challenges in data handling. Smartphone-based systems emerge as a promising solution offering rapid, convenient, and precise detection of Hb concentrations. The adaptation of existing models or the development of novel approaches to refine the segmentation process is crucial for predicting Hb concentrations on mobile devices, aiming for lightweight yet highly effective neural networks. Our research contributes to this burgeoning field by focusing on advanced segmentation techniques and the development of streamlined neural networks.

This study reports on the development of a deep learning-assisted system that predicts hemoglobin concentration using smartphones. Initially, our research team built a prediction model employing a two-stage approach that combined Mask-RCNN [25] for image segmentation and MobileNet for the prediction phase [26]. This model, trained on a dataset comprising 1,124 perioperative eyelid images of patients, demonstrated a mean absolute error (MAE) of approximately 1.5. By employing a smartphone application to capture ocular images and applying a deep neural network trained on a robust dataset of hemoglobin measurements, our system offers a promising alternative to traditional methods. The use of the EGE-Unet model for eyelid segmentation and the DHA(C3AE) model for hemoglobin level prediction represents an innovative step toward enhancing the precision and convenience of hemoglobin testing.

## Methods

### Ethical statement

The study protocol was approved by the institutional ethics committee of the First Affiliated Hospital of Third Military Medical University (also called Army Medical University, KY2021060), on February 20, 2021, and written informed consent was obtained from each patient. The clinical trial was registered on the Chinese Clinical Trial Registry (No. ChiCTR2100044138) on March 11, 2021. The principal researcher was Prof. Bin Yi.

### Patient recruitment and image collection

The patient recruitment and image collection phase were conducted at the First Affiliated Hospital of the Third Military Medical University in Chongqing, China, from March 18, 2021, to April 26, 2021. The study set forth specific inclusion criteria: willingness to participate in the research and capability to adhere to the study protocol; necessity for Arterial Blood Gas (ABG) analysis as part of routine clinical care; and a perioperative Hemoglobin variance exceeding 1.5 g/dL. Conversely, the exclusion criteria encompassed: refusal to participate; incapacity to cooperate due to mental health conditions; presence of eye diseases, exposure to eye or facial radiation therapy; affliction by carbon monoxide or nitrite poisoning, jaundice, or any condition affecting the conjunctiva color; or any other factor deemed by researchers to render a participant unsuitable for the study.

To facilitate patient enrollment, image capture, data collection, and image analysis, a standardized research methodology was established. The research team comprised eight members, each assigned specific roles: one for patient recruitment, two for capturing images, two for data collection and management, one for conjunctiva analysis, and two for quality assurance. Before the commencement of patient recruitment, all team members underwent training to familiarize themselves with the study’s procedures, including the inclusion and exclusion criteria, conjunctiva exposure and image capture techniques, and conjunctiva analysis standards.

On the day preceding surgery, eligible patients who consented to participate signed a written informed consent form. On the day of surgery, following ABG analysis, the designated team members proceeded to the operating room or the post-anesthetic care unit (PACU) to photograph the patients’ right and left facial profiles, ensuring standard conjunctiva exposure under the typical lighting conditions of the operating room and PACU. The interval between the ABG analysis and the image capture did not exceed 10 min. All photographs were taken with the patients in a supine position, using the rear camera of the same smartphone (20.00 megapixel and f/1.8 aperture) under identical settings. Simultaneously, two other team members recorded patient identifiers, gender, Hb levels, age, and other pertinent information.

At the end of each day, the data collection team reviewed the images to identify patients with Hb variations greater than 1.5 g/dL, discarding all unselected images permanently. The quality control team oversaw the entire process, ensuring the integrity of patient recruitment, image quality, and data accuracy throughout the study.

### Workflow and experimental methodology

**Fig. 1:**
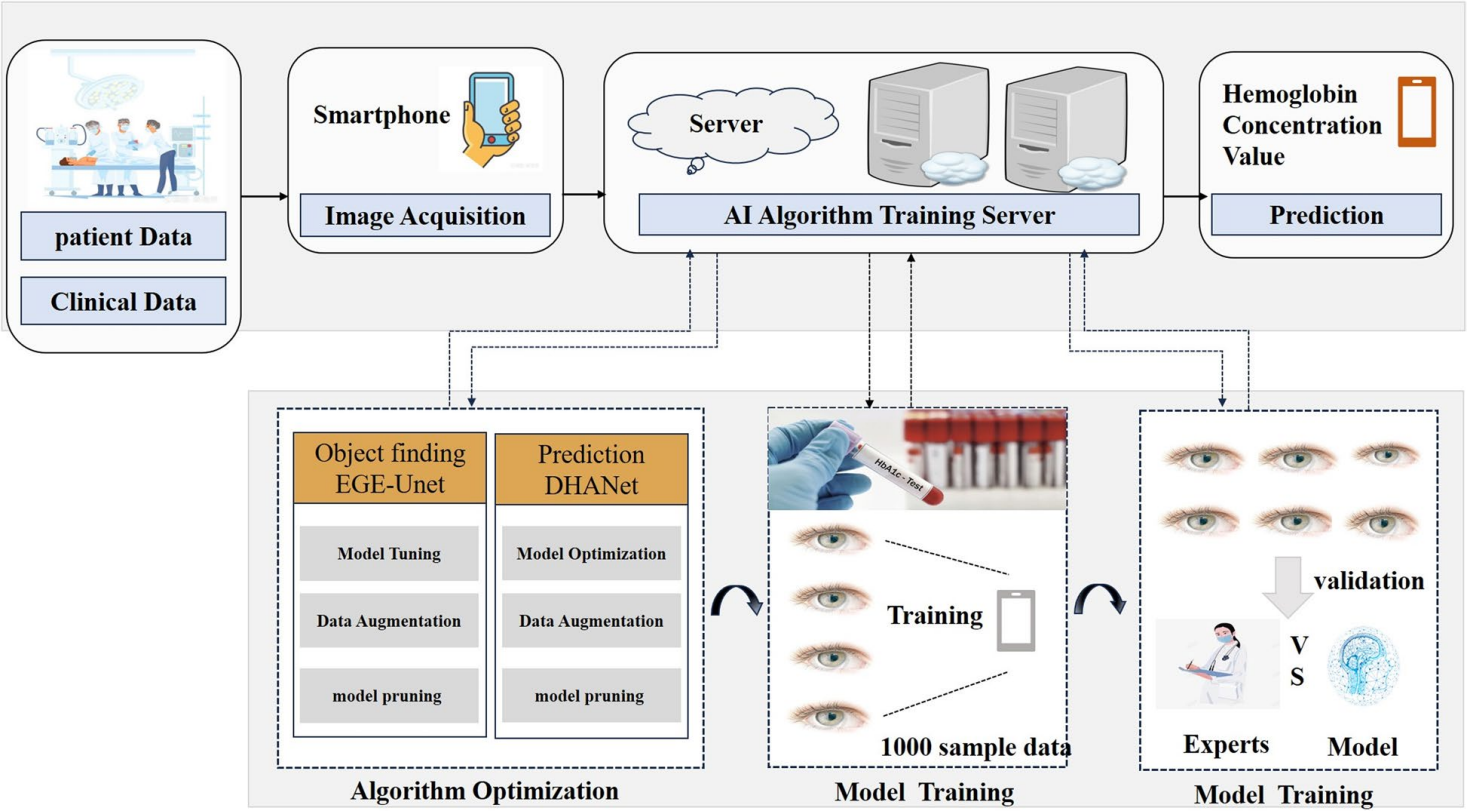
Workflow of model for Non-Invasive Prediction of Hemoglobin

In this research, we innovated a smartphone-based solution capable of estimating hemoglobin levels through the application of deep learning. This system utilizes a smartphone application to capture eye skin images, which are subsequently analyzed by a deep neural network. This network has undergone training on a dataset comprising Hb measurements obtained via invasive blood tests. It employs features extracted from the skin images to forecast Hb concentrations. The system’s workflow and the experimental setup are delineated below. Fig. 1 provides a schematic overview of our system’s workflow and the study’s experimental framework. The system encompasses an algorithm dedicated to eyelid segmentation and another algorithm designed for predicting Hb concentrations based on these values (refer to Fig. 1). Leveraging deep learning technology, we accomplished swift and reliable detection of Hb levels in patients undergoing surgery.

To compile training datasets, we captured eyelid images from patients using various smartphone models. Data augmentation techniques were employed to enhance the deep learning method’s accuracy and robustness. The efficacy of the trained model was assessed on novel datasets, with its precision being verified against a collection of 265 test samples. To further validate the model’s accuracy, we conducted a comparative analysis involving two distinct experimental cohorts: one comprising human experts and the other utilizing the prediction model outlined in this paper, both evaluating the same set of 265 test images. Medical professionals estimated the Hb concentration range based on the patients’ eye images and assessed the accuracy of their estimations. Conversely, the smartphone application processed the eye images through segmentation and subsequently forecasted the Hb values. The application then precisely determined the prediction error within a specified range. This experimental design not only highlights the potential of mobile technology in medical diagnostics but also showcases the accuracy and efficiency of deep learning algorithms in predicting critical health markers such as hemoglobin levels.

### Image data augmentation

**Fig. 2:**
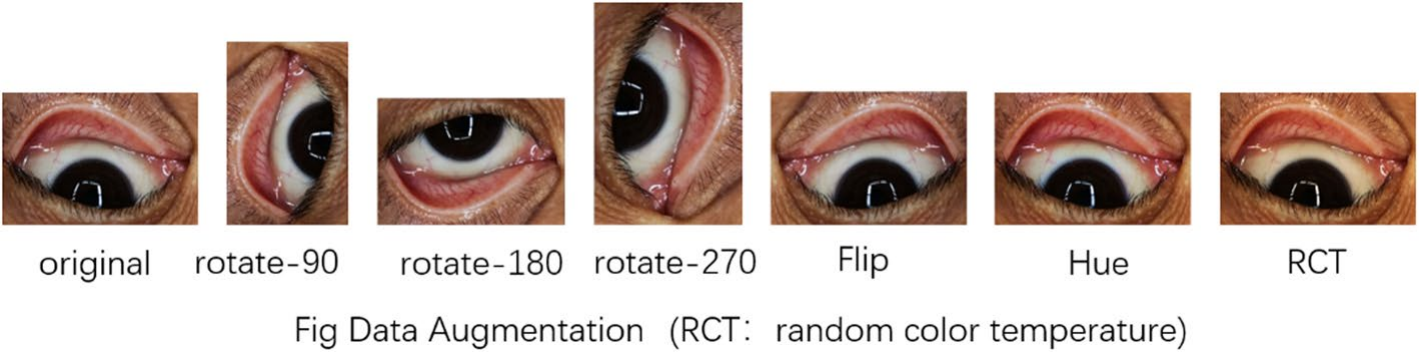
Image regeneration

To effectively train deep learning models, a substantial volume of training data is essential. Nonetheless, enlarging the training dataset poses a significant challenge. A practical approach to augment the volume of training data involves the reproduction of existing data. This process generates multiple images from a single source by randomly applying a combination of techniques illustrated in Fig. 2, which includes: (A) color temperature adjustment, (B) contrast enhancement, (C) brightness alteration, (D) Gaussian blur application, (E) horizontal flipping, and (F) stochastic cropping and resizing. Techniques A, B, and C address the variability in color representation across different smartphone models, ensuring the model is not biased toward the color metrics of a specific device. Technique C also accounts for the diverse lighting conditions under which photos might be taken, ranging from dimly lit environments to brightly illuminated settings. Technique D introduces an element of blur to simulate photos taken out of focus, a common occurrence in hastily captured images. Techniques E and F are designed to mimic minor inaccuracies in framing and alignment that can occur during the photo capture process, ensuring the model can accurately process images despite slight imperfections. By employing these data augmentation techniques, we not only increase the diversity of our training dataset but also enhance the robustness and generalizability of our deep learning model to accurately interpret images under a wide array of conditions typical of smartphone photography.

### Model optimization for precise eyelid detection and hemoglobin concentration prediction

The effectiveness of many AI-based diagnostic methods can significantly diminish when faced with variations in lighting conditions, camera angles, and other external influences. To counteract these challenges and enhance algorithmic performance, this study employed two distinct algorithms: 1) an Eyelid Semantic Segmentation Algorithm, and 2) a Prediction Algorithm based on color intensity analysis. The model's performance was assessed utilizing a deep learning framework, which was implemented directly within a smartphone application, as depicted in Fig. 1. Initially, the Efficient Group Enhanced UNet (EGE-Unet) [27] was deployed to accurately identify the target eyelid regions. The success of the prediction algorithm was found to be closely tied to the precise localization of these regions of interest. Subsequently, the deep learning network was tasked with performing hemoglobin concentration predictions, through which the DHANet model emerged as the superior prediction model due to its exceptional accuracy.

In the concluding phase of model optimization, the DHANet model was selected as the definitive choice for executing highly accurate hemoglobin concentration predictions. This decision was based on its proven efficacy in diagnosing concentration levels accurately, thus underscoring the critical importance of both accurate eyelid segmentation and effective color intensity analysis in enhancing the performance of AI diagnostic tools, especially when operated in the variable and unpredictable environment of smartphone applications.

### Deep learning model architecture

This study introduces a deep learning model structured in two pivotal stages: the Region of Interest (ROI) cropping stage and the decision-making stage. This bifurcation stems from the observation that in diagnostic imaging, particularly as illustrated in Fig. 1, the most informative content is often localized within a small area near the test line. Direct decision-making from the original, full-sized image is inefficient due to the disproportionate ratio of relevant information to the overall image size. This model draws inspiration from human diagnostic practices, where focus is typically narrowed to the test line area. By mimicking this approach—segregating the precise cropping of the test line area (ROI cropping stage) from the diagnostic analysis utilizing the test line's data (decision stage)—we aim to enhance learning efficiency.

The EGE-Unet [27] model, an advanced iteration of the traditional U-Net [28] designed to address challenges in medical image segmentation, is deployed during the ROI cropping phase. It incorporates two novel modules: the Group multi-axis Hadamard Product Attention module (GHPA) and the Group Aggregation Bridge module (GAB). The GHPA module facilitates the extraction of lesion information from various angles by grouping input features and applying Hadamard Product Attention operations across different axes, an idea inspired by the Multi-Head Self-Attention mechanism. Meanwhile, the GAB module merges semantic features and detail features across scales, alongside the masks generated by the decoder, through group aggregation. This integration enables the extraction of multi-scale information efficiently. The EGE-Unet model stands out for its segmentation accuracy, low parameter count, and computational simplicity, making it particularly suited for practical applications.

**Fig. 3:**
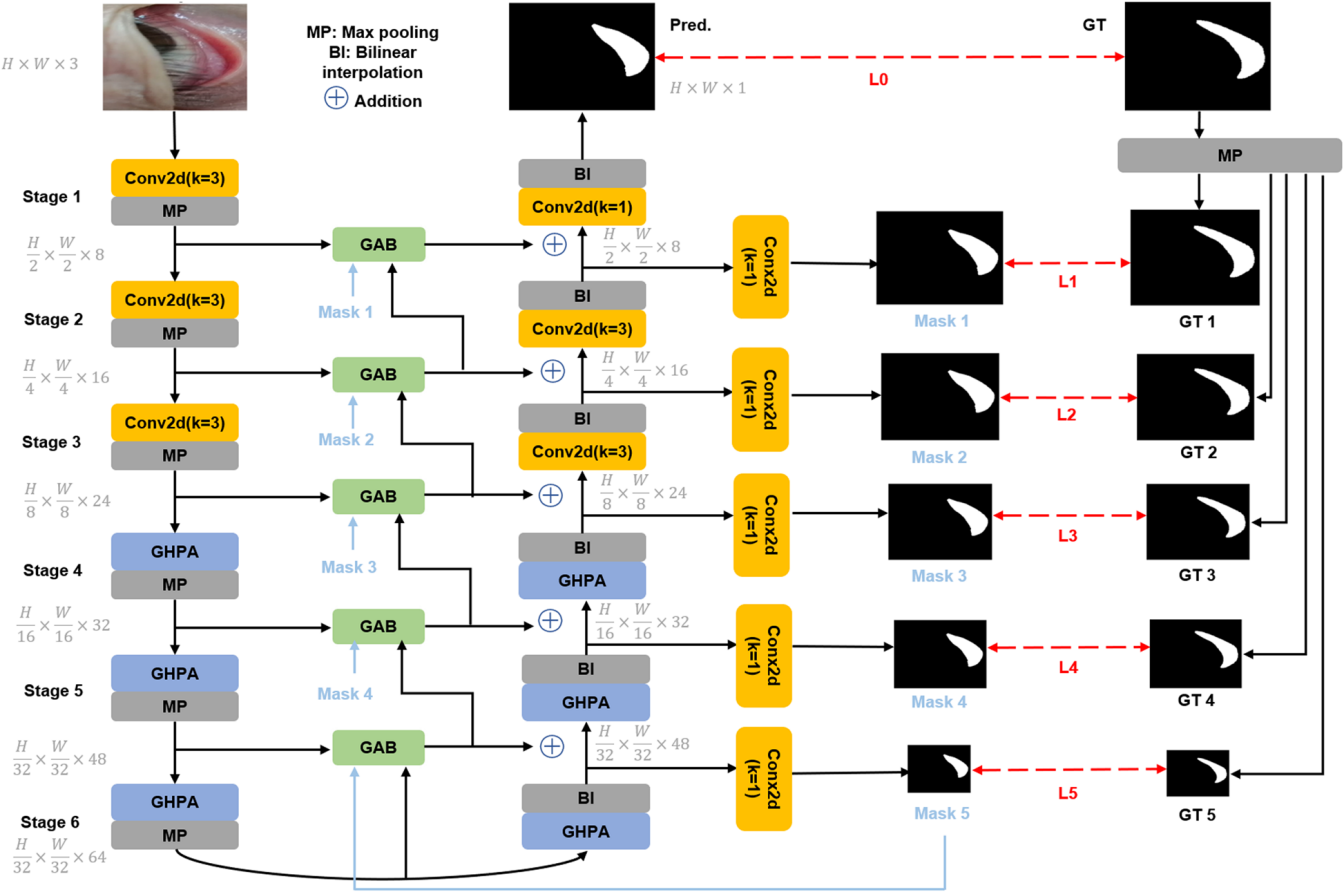
The overview of the segment network

Figure 3 delineates the EGE-UNet's design, showcasing a U-shaped layout with symmetrical encoder-decoder components. The encoder is segmented into six stages, each characterized by varying channel numbers. The initial stages employ standard convolutions, while the latter ones utilize the GHPA for multi-perspective representation extraction. Each encoder-decoder junction incorporates the GAB, enhancing upon the simplistic Skip connections found in the original U-Net. Deep supervision is employed to facilitate mask predictions at multiple scales, contributing to the GAB inputs. These enhancements enable EGE-UNet to surpass previous methods in terms of segmentation efficacy while maintaining a reduced parameter and computational footprint. For an in-depth discussion on the GHPA and GAB modules, refer to reference [27].

**Fig. 4:**
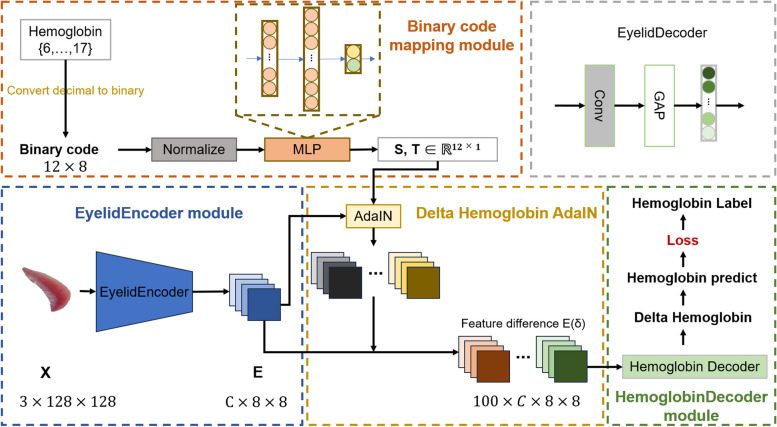
The overview of the prediction network

The decision stage of this paper introduces a hemoglobin concentration prediction model that adopts a regression-based approach. Drawing inspiration from the miniaturized face detection Delta Age AdaIN (DAA) network [29], this method encodes age into binary form for input into a Transfer learning framework to capture continuous age-related feature information. The binary code mapping yields two groups of values corresponding to the mean and standard deviation of the comparison ages, respectively. The age decoder calculates the difference in age, and the mean of all comparisons and difference ages is utilized for age prediction. This methodology is adapted for the eyelid prediction stage, as depicted in Fig. 4.

The architecture of the eyelid prediction system, as depicted in Fig. 4. The top left of the figure shows the eyelid coding section, where the eyelid images for each concentration value (1 unit interval) are encoded in binary 8 bits to form a comparison standard feature; the bottom left of the figure shows the eyelid image feature extraction and conversion with style migration, where the extracted image features are learned from the standard features with difference features; the bottom right of the figure shows the decoding of the difference features to predict the eyelid hemoglobin concentration values; the decoding section of the network structure is located at the top right of the figure. The specific 4 components are as follows: 1) EyelidEncoder Module: This pivotal module transforms the eyelid image into a comprehensive feature vector, encapsulating essential characteristics of the eyelid. For this purpose, the C3AE network [30] is employed due to its efficiency and compactness, making it particularly suitable for deployment on mobile platforms; 2) Delta Hemoglobin AdaIN (DHA): The DHA component is instrumental in estimating hemoglobin concentrations by juxtaposing the current image against a repository of images representing a spectrum of hemoglobin levels. It facilitates hemoglobin concentration prediction by evaluating the feature discrepancies across images; 3) Binary Encoding Mapping Module: Given that hemoglobin concentration variation is a continuous and gradual phenomenon, an 8-bit binary code is utilized to encapsulate the range of hemoglobin concentrations. This method employs binary encoding to transform the continuous spectrum of hemoglobin levels into a discrete, yet seamless, binary representation, enhancing the model's efficiency and interpretability; 4) EyelidDecoder Module: Acting as the final step in the prediction pipeline, the EyelidDecoder module interprets the outputs from both the EyelidEncoder and the binary encoding mapping modules. Utilizing this consolidated information, accurately predicts the patient's hemoglobin concentration levels. The overall algorithm for combining eyelid image segmentation and noninvasive hemoglobin concentration prediction is as follows:

**Table:** 

**Algorithm: Non-invasive Prediction of Hemoglobin**	
1	**// Pseudocode for Automatic Non-Invasive Prediction of Hemoglobin ** **// Using Deep Learning-Assisted Smartphone-Based System**
2	**Initialize:** ** Load trained deep learning model ** ** Initialize smartphone camera settings for optimal image capture** ** Load image preprocessing tools ** ** Load model prediction**
3	**CaptureImage():** ** Capture image using smartphone camera** ** Return image**
4	**PreprocessImage(image):** ** Apply normalization or standardization** ** Resize image to match model input requirements** ** Return preprocessed_image**
5	**SegmentImage(PredictHemoglobin**) ** model_input = format_as_model_input(preprocessed_image)** ** Segment_image=EGE-Unet(model_input)** ** Return Segment_image**
6	**PredictHemoglobin(Segment_image):** ** model_input = format_as_model_input(Segment_image)** ** hemoglobin_level = DHA(C3AE).predict(model_input)** ** Return hemoglobin_level**
7	**DisplayResult(hemoglobin_level):** ** Display hemoglobin level on smartphone screen**
8	**Main:** ** image = CaptureImage()** **preprocessed_image = PreprocessImage(image)** ** segment_image=SegmentImage(preprocessed_image**) ** hemoglobin_level = PredictHemoglobin(segment_image)** ** DisplayResult(hemoglobin_level)**
9	**// End of Pseudocode**

In refining the DHA prediction model, the performance of the EyelidEncoder module was optimized by evaluating the resnet18 network in comparison to the original c3ae model. Additionally, a range of widely recognized mobile image processing architectures—MobileNet [31], MobileNetV2 [32], MobileNetV3 [33], Shufflenetv2 [34], Squeezenet [35], Wideresnet [36], Resnet18_CBAM [37], PFLD [38], and BCNN—were analyzed for their applicability. Notably, the PFLD model is recognized for its compact structure, suitable for age prediction, while the BCNN, a simple 5-layer convolutional network, was developed in-house. The efficacy of these models was assessed using various metrics, including Mean Absolute Error (MAE), Mean-Square Error (MSE), and R-Squared (R2), to ensure a comprehensive evaluation of model performance.

### Experiments on the server

The experiments were conducted using the open-source PyTorch learning framework and programmed in Python. The hardware setup for these experiments was hosted on a Dawning workstation at the Chongqing Institute of Green and Intelligent Technology, part of the Chinese Academy of Sciences. This setup boasted dual NVIDIA 3090 graphics cards, each with 11 GB of memory, and ran on a 64-bit Ubuntu 16.04 operating system.

### Model porting and mobilization

**Fig. 5:**
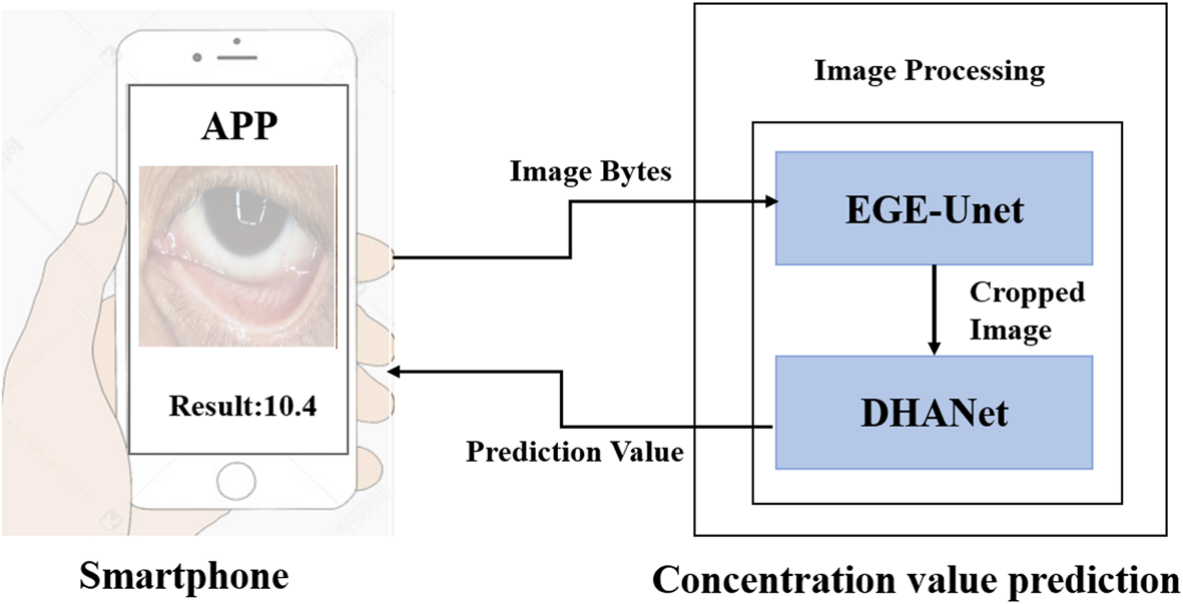
Smartphone application

A smartphone application for Android systems was developed to facilitate hemoglobin concentration estimation directly from eyelid images. As depicted in Fig. 5, the mobile application is divided into two main sections: sampling detection and case management.

Sampling Detection Section: 1) Photo-taking Functionality: Users can capture images using both the front and rear cameras of their device. The application features an interface with a target detection box to guide users in framing the eyelid within the photograph. Alternatively, users can select existing images from their photo album for analysis; 2) Eye Area Image Display: Captured images of the eye area are displayed through the application's interface for review and further processing; 3) Hemoglobin Concentration Recognition: The application employs the developed model to analyze the selected eye area images, determining hemoglobin concentration levels and highlighting specific regions associated with these levels through mask areas; 4) Result Display Function: The detected eyelid area, mask area, and calculated hemoglobin concentration values are presented to the user, enabling easy visualization and understanding of the results.

Case Management Section: Users have the capability to store detection outcomes and enter patient details to create a new case file. Future sampling for the same patient can be added and linked to the existing case, allowing for monitoring of hemoglobin level changes over time.

The model porting process to a mobile platform involves several technical steps. Initially, the segmentation and prediction models are converted to the ONNX format for broader compatibility. Subsequently, model invocation is handled via OpenCV, with inference code crafted in C++. This inference logic is then packaged through NDK cross-compilation, leading to the creation of an SDK with a standardized C interface. The final application interface and related logic are developed in Android Studio, enabling the SDK to perform predictions through a conventional C interface. This comprehensive approach ensures the seamless integration of sophisticated deep learning models into user-friendly mobile applications, enhancing accessibility and utility for end users.

## Results

### Image pre-processing

In this study, we enrolled 284 patients scheduled for elective surgery, including 117 males and 167 females, and collected a total of 1273 eye images. After excluding three images due to inadequate exposure and five due to overexposure, we were left with a dataset of 1265 images. This dataset was divided into 1000 images for training and 265 images for testing. The hemoglobin concentration distribution in the training dataset closely mirrored that of the test dataset, ensuring consistency in model evaluation.

### Segmentation experiment

**Fig. 6:**
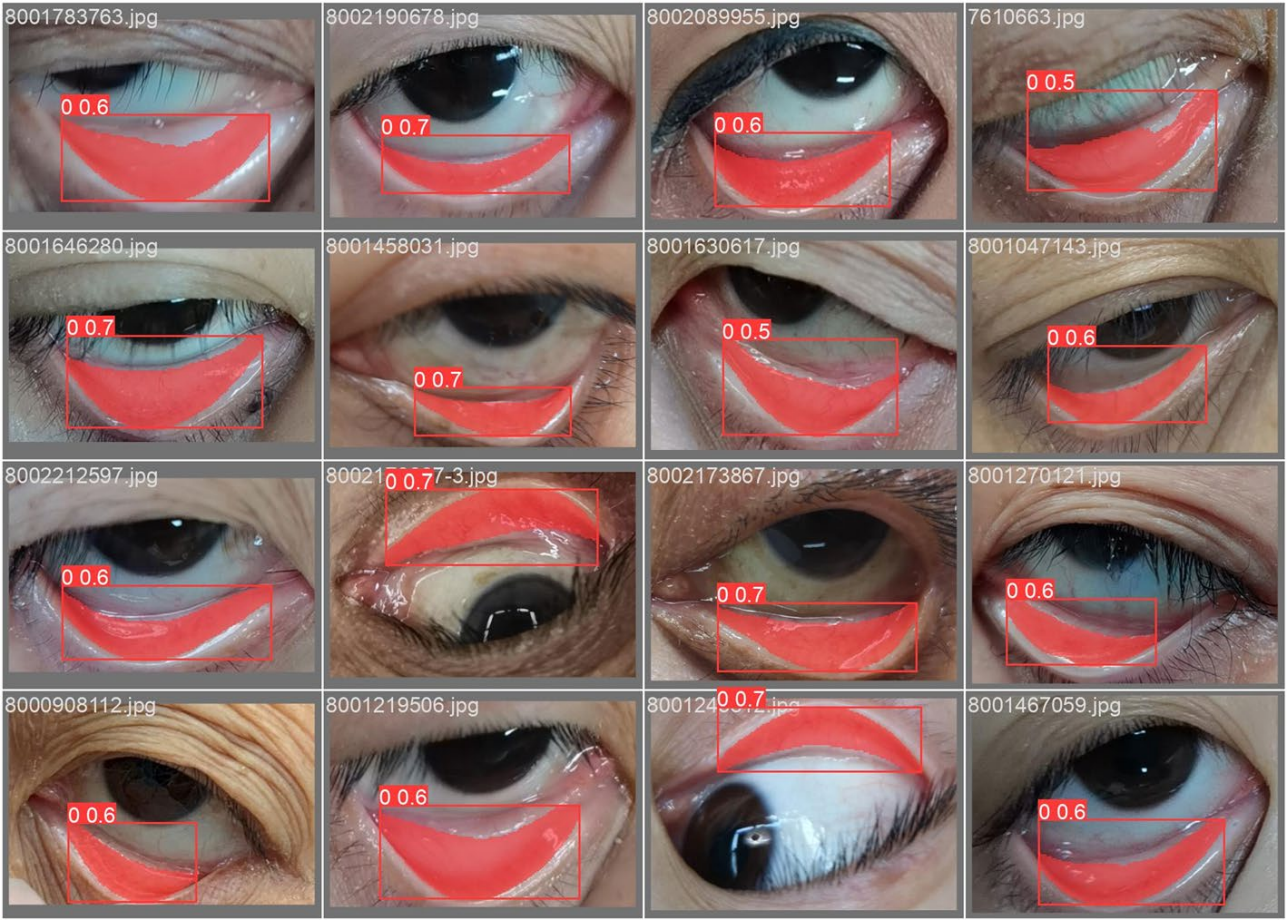
Schematic diagram of segmentation results

For the segmentation task, we utilized the EGE-Unet model, applying multi-scale training to the training set with a batch size of 64. The Stochastic Gradient Descent (SGD) optimizer was used, setting the initial learning rate (LR) at 0.01 over 300 epochs. A StepLR decay strategy was employed, halving the LR at the 16th epoch and doubling it at the 20th epoch. The results of this segmentation experiment are illustrated in Fig. 6, comparing the performance of the proposed model against YOLOv8 [39] and Mask RCNN.

The outcome of this experiment, as detailed in Table 1, revealed that EGE-UNet surpassed the comparative models, achieving Mean Intersection Over Union (MIOU) of 0.78, an F1 Score of 0.87, an accuracy of 0.97, a specificity of 0.98, and a sensitivity of 0.86. Furthermore, Table 2 highlights the efficiency of EGE-UNet: the model parameters totaled only 0.05M, with a computational requirement of merely 0.08 FLOPs (G), and an overall model size of just 0.5M. This performance was on par with that of YOLOv8 and Mask RCNN; however, EGE-UNet's model size was significantly more compact, being 30 times smaller than YOLOv8 and 3000 times smaller than Mask RCNN. Additionally, it required 20 and 50 times less computational complexity, respectively, marking a substantial improvement in model efficiency and practical applicability.

**Table 1:** The segmentation results

Model	MIOU	F1 Score	Accuracy	Specificity	Sensitivity
EGE-UNet	0.78	0.87	0.97	0.98	0.86
YOLOv8 [30]	0.34	0.51	0.92	0.96	0.47
Mask-RCNN [16]	0.67	/	0.98	/	/

**Table 2:** Segmentation Model Metrics

Model	Molde Size(M)	Params(M)	FLOPs(G)
EGE-UNet	0.5	0.05	0.08
YOLOv8 [39]	6.23	3.20	8.70
Mask-RCNN [25]	169	43.92	121.76

### Prediction experiment

In the predictive analysis phase, the Delta Hemoglobin AdaIN (DHA) model was employed as the foundation for training the prediction model. A crucial aspect of this experiment involved adjusting the EyelidEncoder module to evaluate the performance of the resnet18 network against the c3ae model. Concurrently, this study also benchmarked popular mobile image processing models against the DHA model to ascertain their relative performance. The findings from this comparative analysis are summarized in Table 3.

**Table 3:** Prediction result

model	MAE	MSE	RMSE	R2	Mode Size	Params(M)	FLOPs(G)
DHA(C3AE)	1.34	2.85	1.69	0.34	0.58	0.05	0.04
DHA (resnet18)	1.33	2.79	1.67	0.36	129	11.32	2.37
Resnet18_CBAM	1.35	2.86	1.69	0.34	43	11.27	1.77
MobileNet	1.58	4.16	2.04	0.04	12.4	3.23	4.32
MobileNetV2	1.47	3.45	1.85	0.20	8.87	2.26	4.37
MobileNetV3	1.56	3.64	1.90	0.16	4.26	1.85	0.07
Shufflenetv2	1.46	3.33	1.82	0.23	5.03	1.27	4.08
Squeezenet	1.34	2.98	1.72	0.31	2.89	0.74	3.26
Wideresnet	1.41	3.30	1.81	0.24	65.3	17.31	129.47
BCNN	1.52	3.51	1.87	0.19	96.3	25.2	3.58
PFLD	1.52	3.53	1.88	0.19	5.33	1.37	0.11

**Fig. 7:**
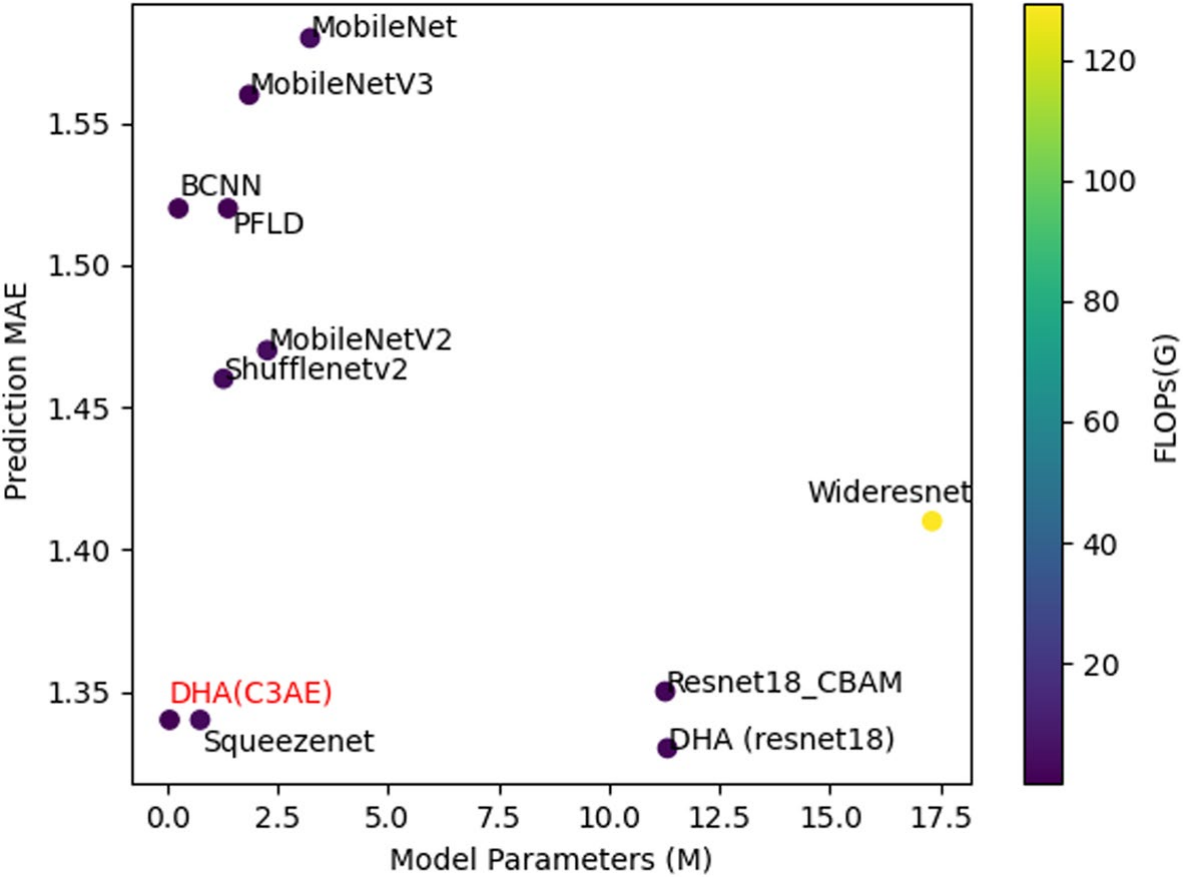
Network Parameter vs. Prediction MAE. Color Depth Representation Model FLOPs (G)

From the data presented in Table 3 and the visual representation in Fig. 7, it is evident that the most effective model configuration was attained when employing C3AE as the EyelidEncoder. This configuration yielded impressive outcomes, characterized by a Mean Absolute Error (MAE) of 1.34, a Mean-Square Error (MSE) of 2.85, a Root Mean Square Error (RMSE) of 1.69, and an R-squared (R2) value of 0.34. Furthermore, the model was distinguished by its minimal parameter size of only 0.05M and a remarkably low computational demand of 0.04G FLOPs. Comparatively, when the EyelidEncoder was configured with resnet18, the model's performance metrics were analogous to those achieved with C3AE. However, the resnet18 configuration necessitated model parameters that were 226 times larger and incurred a computational complexity 59 times greater than that of C3AE. Although mobile network models such as MobileNet, MobileNetV2, MobileNetV3, and Squeezenet demonstrated slightly less optimal performance than the C3AE network, they were characterized by significantly larger parameters and higher computational complexity, except the Squeezenet2 model. Notably, Squeezenet2 achieved an MAE comparable to that of c3ae, yet required model parameters 14 times larger and a computational complexity 81 times greater.

Moreover, the analysis revealed that convolutional networks designed on a simplistic framework exhibited parameter sizes and computational complexities akin to the C3AE network. Nevertheless, their performance metrics did not measure up to those of the C3AE model, underscoring the superior efficiency and efficacy of the C3AE-based EyelidEncoder configuration in hemoglobin concentration prediction tasks. This study highlights the importance of selecting an appropriate EyelidEncoder to balance model performance with operational efficiency, especially in applications intended for mobile platforms.

### Clinical sample test evaluation

**Fig. 8:**
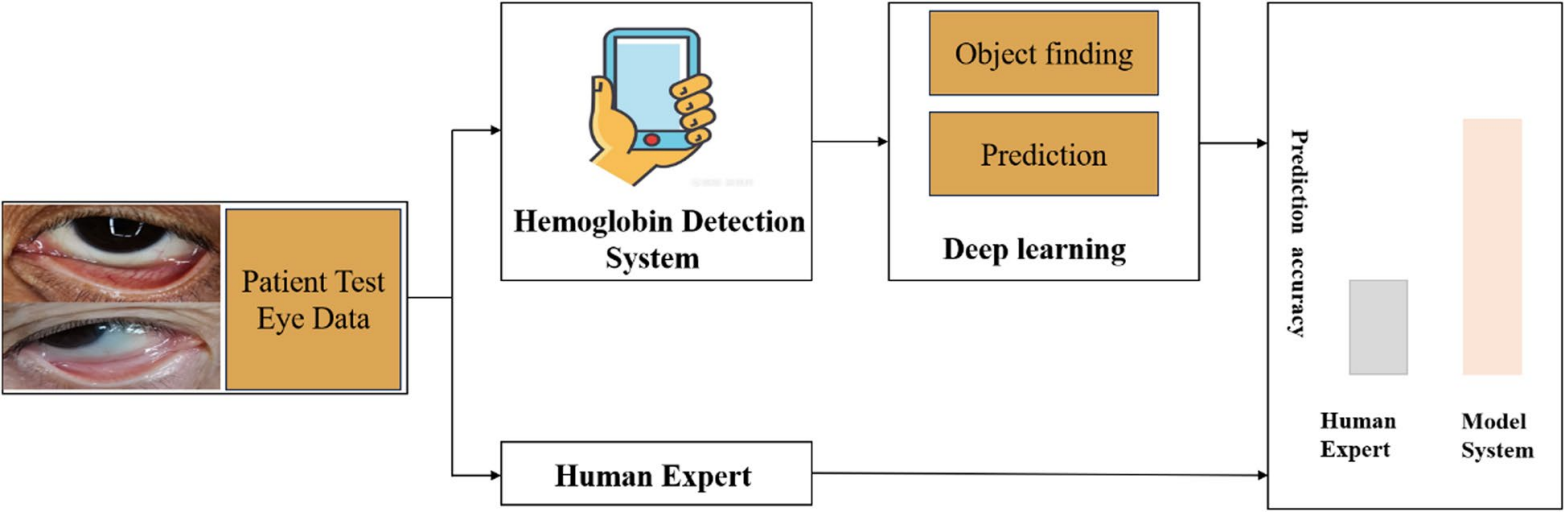
Compare with medical experts

**Fig. 9:**
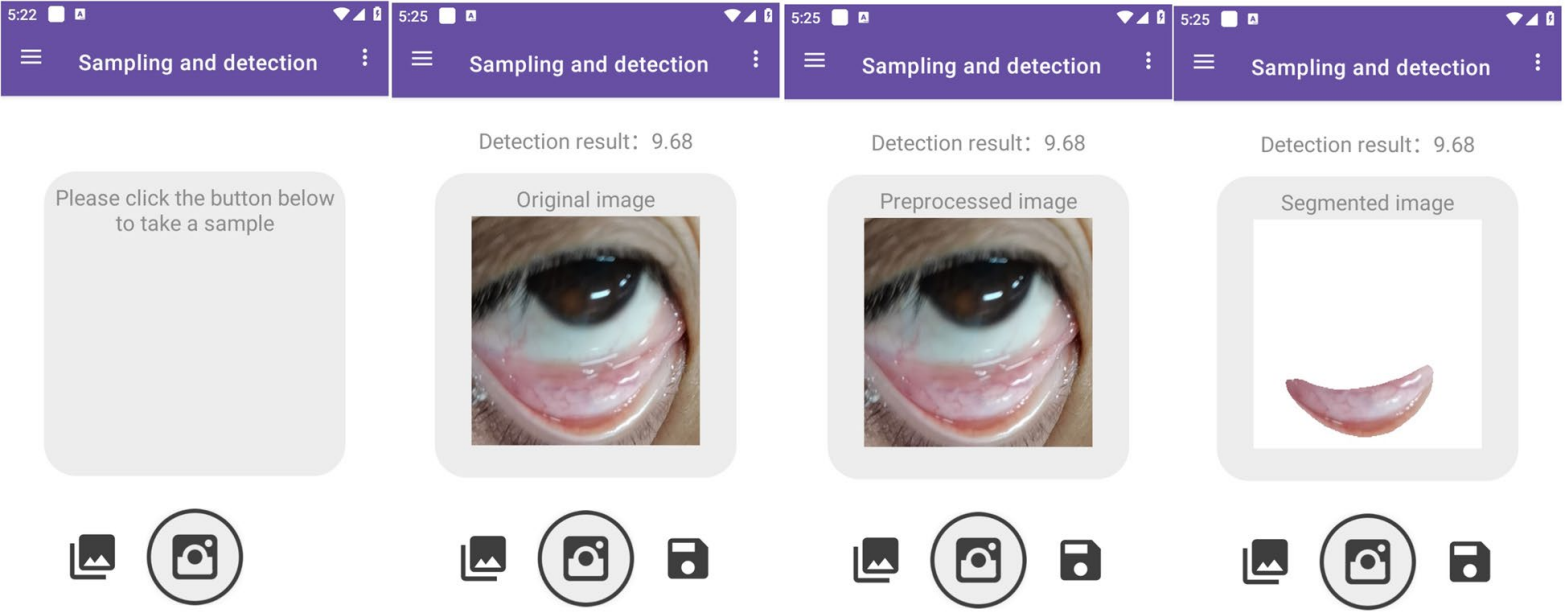
Mobile app detection demonstration

A blind testing approach was utilized to assess the efficacy of the proposed model in predicting hemoglobin concentrations within clinical samples, as depicted in Figs. 8 and 9.

Figure 9 highlights the functionality of a mobile application specifically developed for this purpose. The application's workflow encompasses capturing an image of the patient's eyelid, identifying the relevant eyelid area for segmentation, and subsequently predicting the hemoglobin concentration. The results of this practical application, juxtaposed with the evaluations made by two medical experts and two junior doctors, are showcased in Table 4 Before the assessment, both the experts and junior doctors were familiarized with 1000 trained images, which they used as a reference for evaluating the hemoglobin concentrations based on patients' eyelid images.

**Table 4:** APP VS Expert Prediction Result

Interval	APP(MAE)	Expert-1	Expert-2	Junior doctor-1	Junior doctor-2	Number
6–7	1.28	4.30	0.20	1.00	0.09	2
7–8	1.42	3.83	3.36	2.14	2.14	18
8–9	1.57	2.72	3.29	2.13	2.38	30
9–10	1.29	1.90	2.47	1.98	2.16	48
10–11	1.03	1.13	2.27	2.04	2.26	48
11–12	1.03	0.55	1.66	1.95	2.54	34
12–13	0.99	0.87	1.15	2.71	2.88	36
13–14	1.66	1.66	0.80	3.17	3.37	23
14–15	2.35	2.37	1.21	3.83	4.57	18
15–16	2.60	3.19	1.78	4.21	3.85	6
Average	1.33	1.73	2.30	2.38	2.64	265

According to Table 4, the optimal Mean Absolute Error (MAE) estimated by the medical experts was 1.73, while the mobile application demonstrated a significantly improved accuracy with an MAE of 1.33, marking a 23% enhancement in accuracy over the experts' predictions. Additionally, the distribution of prediction errors by the app closely mirrored that of the experts, with minimal estimation biases observed in patients with normal values and larger biases in patients exhibiting abnormal values at both extremes of the spectrum. Conversely, the MAE observed in junior doctors was 2.64, reflecting a notable deficit in prediction accuracy attributable to their comparative lack of experience.

Error metrics detailed in Table 5 range from 0 to 2.0, reinforcing the reliability of predictions made by both the mobile application and the experts. As delineated in Table 5, the mobile application achieved a 60% accuracy rate at an error range of 1.5, surpassing the experts' accuracy rate of 55%. In contrast, the accuracy rate of junior doctors was notably lower, plummeting to 48% at an error range of 2.0. These results underscore the potential of the mobile application to significantly aid physicians in enhancing the real-time accuracy of hemoglobin concentration predictions in patients, thereby contributing to more informed and effective clinical decision-making.

**Table 5:** Comparison of predictions between different intervals

Interval ranges	APP accuracy	Expert-1	Expert-2	Junior doctor-1	Junior doctor-2
1	0.44	0.35	0.32	0.27	0.25
1.5	0.60	0.55	0.48	0.35	0.33
1.8	0.70	0.62	0.53	0.41	0.39
2.0	0.75	0.66	0.57	0.48	0.46

## Discussion

In this project, we developed an innovative smartphone-based application for the non-invasive prediction of hemoglobin concentration, utilizing a sample-to-hemoglobin concentration strategy coupled with deep learning to facilitate decision-making. This application leverages patient-derived eyelid images captured through smartphone cameras and employs a micro-network framework, achieving results with minimal computational demand, at only 0.12 FLOPs(G). The predictive accuracy of the application shows promise for enhancement through training with additional clinical data. Its efficacy and versatility were confirmed through extensive testing across multiple users and prediction models, establishing its utility as a tool for detecting hemoglobin concentration.

Our approach, which leverages mobile phone-captured eyelid photos for estimating hemoglobin levels, presents a convenient and cost-effective method compared to previous studies [20]. Unlike other research focusing on conjunctival judgment for anemia diagnosis [22, 40], our model offers quantitative hemoglobin level detection through non-invasive conjunctival examination. A critical aspect of our research is the blind test, which compares the predictions made by the mobile application with those of medical experts. The superior accuracy of our system, as indicated by a lower MAE, underscores its potential utility for healthcare professionals, particularly in time-sensitive scenarios such as intraoperative massive bleeding where rapid, non-invasive hemoglobin measurement is imperative. Our proposed system's ability to operate on standard smartphone devices positions it as a scalable solution for global health monitoring. The requirement for minimal additional hardware makes it an economically viable option for low-resource settings, where traditional hemoglobin testing methods may be cost-prohibitive.

The application of deep neural networks and their variants in predicting hemoglobin concentration from eyelid images [26, 41, 42] is well-documented. However, the extensive parameters and computational complexities of these models restrict their practicality in mobile medical applications. To overcome these challenges, our study introduces a two-pronged solution. Firstly, we employ the UNet (EGE-UNet) for precise segmentation of the patient's eyelid region. The EGE-UNet, integrating the lightweight Group multi-axis Hadamard Product Attention (GHPA) module and Group Aggregation Bridge (GAB) module, excels in extracting and integrating multi-scale information for accurate eyelid segmentation. This model's efficacy and efficiency, as evidenced by its performance against alternatives like YOLOv8 and Mask RCNN and its low computational requirements, make it ideal for smartphone deployment. Secondly, the Delta Hemoglobin AdaIN (DHA) operation is utilized for deriving a representative eyelid image indicative of the patient's hemoglobin concentration through transfer learning. The DHA, a compact yet potent feature learning network, leverages binary encoding to ensure the continuity of feature information, thereby enhancing prediction accuracy. Our findings reveal the smartphone-based system's capability, assisted by deep learning algorithms, to non-invasively predict hemoglobin levels with promising accuracy. Our system relies on ubiquitous smartphone technology and the absence of the need for sophisticated laboratory equipment. We anticipate that the reduced cost of hemoglobin testing will not only benefit healthcare providers through lower operational expenses but also empower patients by making frequent health assessments more affordable. While promising, this study acknowledges limitations such as the relatively small sample size and the necessity for further validation across larger datasets. We acknowledge the potential challenges, such as variability in smartphone camera quality, lighting conditions, and user handling. Future research will address these by developing a standardized protocol for image capture and by enhancing the model's robustness to different environmental factors. In addition, future work will focus on refining the deep learning models to improve accuracy and reduce false predictions. Meanwhile, integrating real-time feedback mechanisms into the smartphone application could enhance user engagement and provide immediate guidance on potential health concerns. We are also considering the inclusion of a feature that tracks hemoglobin levels over time to identify trends and alert users to significant changes. For practical applications, it is necessary to conduct large-scale clinical trials to validate our system's effectiveness in real-world settings. This will involve collaboration with healthcare providers to integrate our technology into routine check-ups and remote patient monitoring programs.

## Conclusions

In summary, our research demonstrates the feasibility of non-invasively predicting hemoglobin levels using a deep learning-assisted smartphone application. This breakthrough has the potential to transform hemoglobin measurement practices, offering a more efficient and patient-friendly alternative for the diagnosis and monitoring of various health conditions. Future studies are essential to validate our results further and refine the system for widespread clinical application. Several aspects require further exploration and validation before this technology can be widely adopted in clinical settings. These include:

1. Large-scale Validation: While our initial results are promising, it is crucial to validate our findings on a larger and more diverse population. This will help ensure that the application is accurate and reliable across different demographics, ages, and health conditions.
2. Refinement of Algorithms: The deep learning algorithms used in our application need to be continuously refined and optimized. This includes improving the accuracy of predictions, reducing the possibility of false positives or negatives, and enhancing the user interface for better patient experience.

## Data Availability

The datasets used and analyzed during the current study are available from the corresponding author on reasonable request.

## Code availability

The overall source codes used in this study are available at: (https://github.com/keyan2017/ANPHSP).
